# Genetic elastic fiber disorders as neurovascular syndromes: An emerging perspective

**DOI:** 10.4103/NRR.NRR-D-25-01159

**Published:** 2026-01-02

**Authors:** Francesc Jiménez-Altayó

**Affiliations:** Department of Pharmacology, Therapeutics, and Toxicology, School of Medicine, Universitat Autònoma de Barcelona, Cerdanyola del Vallès, Spain; Institute of Neurosciences, Universitat Autònoma de Barcelona, Cerdanyola del Vallès, Spain; Centro de Investigación Biomédica en Red de Enfermedades Cardiovasculares (CIBERCV), Instituto de Salud Carlos III, Madrid, Spain

Genetic elastic fiber diseases arise from inherited or *de novo* mutations in genes encoding elastic fiber components, such as elastin, fibrillin-1, and associated proteins, leading to abnormalities in their deposition, structure, or degradation (Heinz, 2021). Historically, research has focused on systemic, non-neurological manifestations, which are more clinically apparent and often life-threatening, particularly cardiovascular complications. In contrast, potential involvement of the central nervous system (CNS) has received limited attention, even though the brain and spinal cord are richly vascularized structures, extensively perfused, and critically dependent on the integrity of their blood vessels.

Observations of neurovascular complications in broader connective tissue disorders, including autoimmune diseases such as lupus and scleroderma, as well as hereditary conditions such as Ehlers-Danlos syndrome, have highlighted potential neurovascular involvement in related disorders. Yet despite growing awareness and emerging clinical interest (e.g., Parlapiano et al., 2020), the neurovascular and neurological dimensions of elastic fiber diseases remain underexplored. Here, we highlight key genetic elastic fiber diseases with CNS effects, such as Williams-Beuren syndrome (WBS), Loeys-Dietz syndrome (LDS), and Pseudoxanthoma Elasticum (PXE). A notable addition to this group is Marfan syndrome (MFS). Growing clinical and preclinical evidence highlights neurovascular complications and vulnerability in MFS. We argue that recognizing neurovascular risks in genetic elastic fiber disorders is crucial for guiding future therapies and may also provide insights into mechanisms underlying more common neurovascular and neurodegenerative conditions.

**Importance of elastic fibers in the central nervous system:** Elastic fibers are specialized components of the extracellular matrix (ECM), primarily composed of a central core of elastin surrounded by a sheath of fibrillin-rich microfibrils and associated glycoproteins (Heinz, 2021). This structure provides tissues with elasticity and resilience. Within the broader ECM, elastic fibers are embedded in a network that includes structured proteins such as collagens and laminins, as well as amorphous components such as hyaluronan, proteoglycans, and tenascins. These elements interact with cells via surface receptors, facilitating not only structural integrity but also signaling functions critical for tissue maintenance and repair.

Beyond their recognized role in providing elasticity and structural support to blood vessels, though less abundant, elastic fibers within the brain and spinal cord parenchyma, as well as in perivascular and interstitial spaces, contribute to the mechanical stability of the ECM surrounding neural cells. They help shape microenvironments that facilitate neuronal and glial interactions and support cell adhesion and migration. These fibers are also involved in key processes during brain and spinal cord development and are increasingly implicated in various neurological disorders. Among these, transforming growth factor (TGF)-β signaling and interactions with the ECM serve as important bridges connecting vascular stability with neural integrity.

Loss of elasticity can compromise the structural integrity of cerebral and spinal vessels. This leads to dysfunction within the neurovascular unit and impaired regulation of blood flow. White matter is particularly susceptible to these disturbances, as it relies heavily on consistent perfusion and tightly controlled neurovascular coupling. In addition, degradation of elastic fibers may contribute to disruption of the blood–brain barrier (BBB) and the blood-spinal cord barrier, critical defenses that maintain CNS homeostasis. Impaired barrier integrity can exacerbate neuroinflammation and promote neural injury. Taken together, the role of elastic fibers in maintaining the balance between vascular function and neural health is crucial.

**Key syndromes and their emerging neurovascular profiles:** Several genetic disorders involving elastic fiber abnormalities have been linked to neurological and neurovascular complications. Here, we briefly highlight four representative conditions, WBS, LDS, PXE, and MFS, which collectively illustrate the range of CNS manifestations associated with genetically determined elastic fiber pathology. Emphasis is placed on MFS, considering emerging evidence suggesting neurovascular involvement in this syndrome.

**Williams-Beuren syndrome (OMIM 194050):** It is a rare multisystem genetic disorder with an estimated prevalence of 1 in 7,500 live births. It affects multiple organ systems, including the cardiovascular, gastrointestinal, endocrine, and CNS (Kozel et al., 2021). WBS results from a hemizygous deletion of approximately 25–27 genes on chromosome 7q11.23. Among the deleted genes, the elastin gene (*ELN*) is considered a major contributor to the cardiovascular phenotype, particularly the stenosis of large arteries such as the supravalvular aortic and pulmonary arteries.

**Figure 1:**
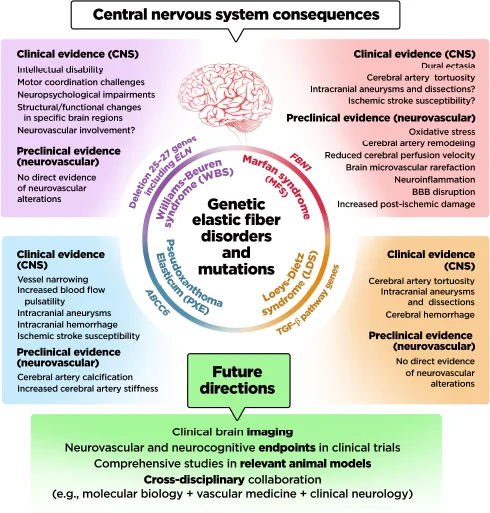
Central nervous system consequences of genetic elastic fiber disorders. This figure summarizes some of the current clinical and preclinical evidence for central nervous system involvement across four major genetic elastic fiber disorders: WBS, LDS, PXE, and MFS. Each condition is linked to specific gene mutations affecting elastic fibers. While preclinical neurovascular alterations are recognized in PXE and MFS, direct preclinical evidence remains lacking for WBS and LDS. Future research should prioritize systematic brain imaging, integration of neurovascular and neurocognitive endpoints in clinical trials, comprehensive studies using relevant animal models, and cross-disciplinary collaborations to elucidate shared mechanisms and therapeutic targets. ABCC6: ATP-binding cassette sub-family C member 6 gene; BBB: blood–brain barrier; ELN: elastin gene; FBN1: fibrillin-1 gene; LDS: Loeys-Dietz syndrome; MFS: Marfan syndrome; PXE: Pseudoxanthoma Elasticum; TGF-β: transforming growth factor-β; WBS: Williams-Beuren syndrome.

Neurologically, individuals with WBS often present with mild to moderate intellectual disability, motor coordination challenges, and neuropsychological impairments that impact social functioning (Kozel et al., 2021). While imaging studies have identified structural and functional changes in specific brain regions (Garvey et al., 2024), it remains unclear whether alterations in cerebral arterial function contribute meaningfully to these neurological features. This raises the possibility of a link between cerebrovascular dysregulation and impaired brain function. Focused mechanistic studies investigating potential neurovascular alterations in the brain and spinal cord of animal models of WBS are warranted and could substantially advance our understanding of neurological outcomes (**Figure 1**).

**Loeys-Dietz syndrome (OMIM 609192):** It is a rare genetic disorder with an estimated prevalence of approximately 1 in 50,000 individuals. It is caused by mutations in *SMAD2* (small mother against decapentaplegic family member 2), *SMAD3* (small mother against decapentaplegic family member 3), *TGFB2* (TGF-β 2), *TGFB3* (TGF-β 3), *TGFBR1* (TGF-β receptor type 1), *TGFBR2* (TGF-β receptor type 2), *IPO8* (importin 8), and likely other genes yet to be identified (Loeys and Dietz, 2024). All except *IPO8* are inherited in an autosomal dominant manner. These mutations impair the TGF-β signaling pathway, which is essential for maintaining ECM integrity and the proper formation of elastic fibers. The syndrome manifests across multiple systems, with vascular, skeletal, craniofacial, cutaneous, allergic/inflammatory, and ocular involvement being the most common. Among these, the greatest clinical concern is the elevated risk of life-threatening vascular complications, especially thoracic aortic aneurysms and dissections (Loeys and Dietz, 2024).

LDS patients may develop arterial tortuosity, aneurysms, and dissections affecting multiple vascular territories, including the posterior circulation, with reports of involvement in the vertebral, carotid, and intracranial arteries, as well as cases of cerebral hemorrhage, underscoring significant neurovascular involvement (Parlapiano et al., 2020; Loeys and Dietz, 2024). Existing preclinical models of LDS should be extended to include systematic evaluation of cerebral and spinal cord vascular territories, which could help uncover the mechanisms underlying neurovascular manifestations (**Figure 1**).

**Pseudoxanthoma Elasticum (OMIM 264800):** It is an autosomal recessive rare genetic disease with an estimated prevalence ranging from 1 in 25,000 to 1 in 100,000 individuals. It is caused primarily by mutations in the *ABCC6* (ATP-binding cassette sub-family C member 6) gene, which encodes a transmembrane ATP-binding cassette transporter (Terry and Uitto, 2020). This condition impacts the elastic fibers found in the skin, eyes, and the cardiovascular and gastrointestinal systems. The most common cause of long-term complications and disability is vision loss, primarily resulting from subretinal neovascularization and degeneration of the macula (Terry and Uitto, 2020).

Neurovascular complications in individuals with PXE were first described long time ago and are primarily linked to underlying vascular abnormalities, often influenced by renovascular hypertension and associated with conditions such as intracranial aneurysm, intracranial hemorrhage, and ischemic stroke. One of the contributing mechanisms involves progressive fragmentation and calcification of vascular elastic fibers, which affects medium-sized and small intracranial arteries, and can lead to vessel narrowing and increased arterial flow pulsatility. *ABCC6* deficiency in animal models is associated, among others, with cerebral artery calcification and increased cerebral artery stiffness. Notably, investigations using mouse models of PXE, such as *Abcc6*^–/–^ mice, should aim to thoroughly evaluate parenchymal changes in the brain and spinal cord, neurovascular alterations, and explore potential direct neurological consequences, including neurodegeneration (**Figure 1**).

**Marfan syndrome (OMIM 154700):** It is an autosomal dominant rare genetic disorder mostly caused by mutations in the *FBN1* gene, which encodes fibrillin-1, a crucial component of extracellular microfibrils that supports elastic and non-elastic connective tissues. It affects 1 in 5000–10,000 individuals and is characterized by dislocation of the ocular lens, arachnodactyly, abnormal histopathology of the aortic wall, and dilatation of the proximal aorta associated with aortic regurgitation and dissection of the ascending aorta, the latter two being life-threatening (Pyeritz, 2019). The disease pathophysiology includes ECM disorganization, fragmentation of medial elastic fibers, and chronic activation of TGF-β signaling due to impaired sequestration by dysfunctional fibrillin-1. Notably, new features of MFS have emerged since life expectancy has begun to approach that of the general population, mainly because of effective medical therapy and, even more significantly, prophylactic aortic surgery (Pyeritz, 2019).

Although cardiovascular manifestations remain the defining features of MFS, growing evidence points to possible involvement of the cerebrovascular system. The most recognized nervous system feature in MFS is dural ectasia, yet several reports suggest a broader spectrum of vascular changes affecting the brain. These include an increased tendency toward abnormal brain vessel tortuosity, intracranial aneurysms and dissections, and stroke-like events (Parlapiano et al., 2020). Nevertheless, interpretation of earlier studies is complicated by the inclusion of patients who may have had overlapping syndromes, such as LDS. Despite these limitations, tortuosity of the carotid and vertebral arteries has been consistently documented in genetically confirmed MFS patients and appears to be linked to a higher risk of arterial dissection (Parlapiano et al., 2020). The question of whether MFS truly confers a greater risk for intracranial aneurysms and strokes remains unresolved, with recent clinical studies presenting opposing conclusions. Resolving this uncertainty will require well-controlled clinical studies, ideally with genetic profiling of *FBN1* variants, to determine whether specific mutations correlate with cerebrovascular vulnerability. In addition, correlations of these findings with psychiatric and neuropsychological issues identified in some studies are also necessary.

Recent investigations using the *Fbn1*^*C1041G/+*^ mouse model (previously referred to as *Fbn1*^*C1039G/+*^) have begun to uncover neurovascular changes that extend beyond the well-characterized aortic pathology of MFS. Observed alterations include oxidative stress and vascular remodeling in both the middle cerebral artery (Onetti et al., 2016) and the basilar artery (Manich et al., 2025), along with reduced perfusion velocity in the posterior cerebral artery (Curry et al., 2024), potential brain microvascular rarefaction (Curry-Koski et al., 2025), heightened neuroinflammation and BBB disruption (Curry-Koski et al., 2025; Manich et al., 2025), and increased post-ischemic brain damage (Manich et al., 2025). Moreover, when this fibrillin-1 mutation was combined with apolipoprotein E deficiency (*ApoE*^*–/–*^*Fbn1*^*C1041G/+*^) and challenged with a Western-type diet, mice showed increased vulnerability to cerebral injury (Van der Donckt et al., 2015). Altogether, the existing evidence suggests that MFS may be associated with accelerated cerebrovascular aging, potentially making the brain more prone to damage even in the absence of traditional stroke risk factors.

Although these findings are promising, comprehensive studies of both brain parenchyma and cerebral vasculature in other MFS mouse models are still lacking. So far, studies in *Fbn1*^*C1041G/+*^ mice indicate that both sexes display sustained neuroinflammation at early stages (Curry-Koski et al., 2025; Manich et al., 2025) and demonstrate increased sensitivity to ischemic injury, including enhanced post-ischemic neurodegeneration, heightened neuroinflammation, and poorer behavioral outcomes (Manich et al., 2025). In contrast, cerebral artery phenotypes appear to diverge between males and females (Curry et al., 2024; Manich et al., 2025) and vary with age (Onetti et al., 2016; Manich et al., 2025). These findings underscore the importance of age- and sex-stratified analyses to better understand neurovascular function in MFS models. Furthermore, it is critical to expand neurovascular assessments to additional animal models of MFS that represent diverse aspects of the disease, allowing for a more complete understanding of brain-related vulnerabilities. Expanding research beyond the aorta to include both cerebral and spinal cord outcomes may reveal previously overlooked risks and guide clinical management (**Figure 1**).

**Figure 2:**
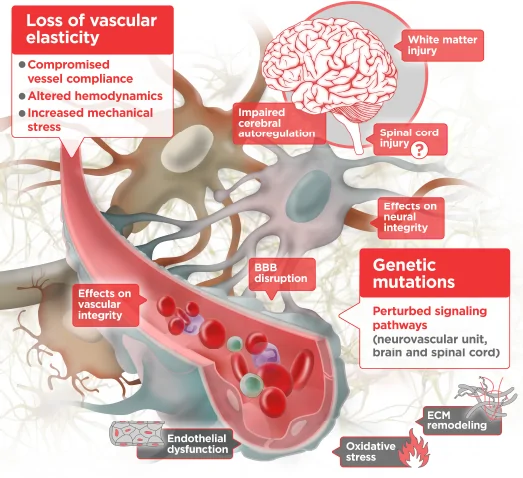
Proposed shared mechanisms linking genetic elastic fiber disorders to neurovascular dysfunction. Loss of vascular elasticity and altered hemodynamics, together with direct genetic effects on signaling within the neurovascular unit, converge to impair vascular and neural integrity. These processes compromise cerebral autoregulation, promote blood–brain barrier (BBB) disruption, and create conditions that increase susceptibility to white matter injury in the brain and possibly the spinal cord. Oxidative stress, endothelial dysfunction, and extracellular matrix (ECM) remodeling may further amplify these effects, contributing to progressive neural vulnerability.

**Potential shared mechanistic pathways:** Although each genetic elastic fiber disorder has distinct molecular origins, the evidence outlined above suggests a convergent cascade underlying neurovascular manifestations (**Figure 2**). Loss of vascular elasticity compromises vessel compliance, leading to altered hemodynamics and increased mechanical stress, while genetic mutations may also directly perturb signaling pathways within the neurovascular unit of the brain and spinal cord. These hemodynamic and genetic factors affect vascular and neural integrity, impairing cerebral autoregulation and promoting BBB disruption. They may also impact the spinal cord, although evidence remains scarce. Together, these events create a permissive environment for white matter injury in the brain and, potentially, the spinal cord. Oxidative stress, endothelial dysfunction, and ECM remodeling may further amplify these effects, contributing to progressive neural vulnerability. Overall, this framework provides a unifying perspective to interpret observations across genetic elastic fiber disorders, linking vascular defects to CNS dysfunction.

**Conclusion and future directions (Figure 1):** The expanding body of evidence calls for systematic CNS imaging in patients with genetic elastic fiber disorders, especially those with neurological symptoms or atypical vascular findings, as well as the inclusion of neurovascular and neurocognitive endpoints in clinical trials. In parallel, comprehensive studies in relevant animal models are essential to uncover disease mechanisms. Overall, these disorders should be reframed as CNS syndromes marked by disrupted vascular and parenchymal integrity in the brain and, potentially, the spinal cord, altered neurophysiological responses, and compromised repair mechanisms. Cross-disciplinary research linking ECM biology, vascular medicine, and clinical neurology may uncover shared pathways driving dysfunction. Focusing more on neurovascular aspects will enhance clinical understanding and inform future therapeutic strategies. Though rare, genetic elastic fiber disorders offer a unique window into neurovascular dysfunction and may help uncover mechanisms that also play a role in stroke, cognitive impairment, and neurodegenerative conditions.

*We thank all researchers in the field and apologize to those whose work was not cited due to space constraints and the guidelines for perspective manuscripts. We are grateful to JC Balasch (Universitat Autònoma de Barcelona, Spain) for the design and preparation of the figures in this manuscript.*

*This work was supported by the Ministerio de Ciencia e Innovación and Agencia Estatal de Investigación of Spain [PID2020-113634RB-C22/AEI/10.13039/501100011033]* *and the Generalitat de Catalunya [2021 SGR 00969] (to FJA).*
